# Editorial: Pain and dementia

**DOI:** 10.3389/fpain.2022.1110495

**Published:** 2023-01-04

**Authors:** Stefan Lautenbacher

**Affiliations:** Bamberg Living Lab Dementia (BamLiD), University of Bamberg, Bamberg, Germany

**Keywords:** pain, dementia, behavior, brain imaging, ethnicity, race

**Editorial on the Research Topic** Pain and dementia

The days when “pain in persons with dementia” was a neglected thematic topic both in dementia and in pain research are fortunately over for more than 10 to 15 years. We can now already consider a diversity of topics and do not only repeat the main message that persons with dementia suffer unnecessarily because nobody cares about their pain. We can meanwhile become topically much more specific. It is therefore still an honour but also already a result of a common trend that *Frontiers in Pain Research* allowed five topic editors (Keela Herr, Bettina S. Husebo, Stefan Lautenbacher, Todd B. Monroe, Catherine Riffin) to set up the Research Topic *Pain and Dementia* for publication. We launched a call to the many experts meanwhile active in that field and were soon successful in recruiting the necessary number of authors.

Non-cognitive symptoms of dementia have been largely overlooked for quite a while but have lately gained more and more interest under the headings “behavioural and psychological symptoms of dementia” (BPSD) or “neuropsychiatric symptoms”. Meanwhile these mainly behavioral problems (e.g., disturbed sleep, aggression, agitation, depression, eating disorders, chronic pain) have been found to be even more burdensome than many of the cognitive problems to the patients, relatives and caregivers.

The present article by Lukas et al. “*Responsive behaviours and pain management in hospital dementia care: a before and after comparison of the ‘Serial Trial Intervention’”* showed a first time attempt to treat BPSD (including pain) in an acute hospital setting by a personalized short-term treatment, namely “Serial Trial Intervention”, without major success, showing the therapeutical resistance of these behavioral problems at least at the short run. However, the authors observed at least more professional awareness of the problems after treatment.

One of the most prevalent and plaguing BPSD symptoms is agitation, which is likely strongly associated with pain. To learn more about the individual relationship between agitation and pain Husebo et al. tested a new algorithm and report in their article “*Understanding pain and agitation through system analysis algorithms in people with dementia. A novel explorative approach by the DIGI.PAIN study”* about the findings. Without finally solving the chicken-egg-problem for the two variables, it becomes clear that strongly related changes of agitation and pain over time are common. However, other temporal relationships may also occur.

Now, the next thematic focus with two articles is highlighted, which is the pain-related functional brain imaging in dementia. Brain imaging is without any doubt very useful for a better understanding of pain processing.

Persons with dementia have often certain cognitive and non-cognitive problems, which make it difficult at large to participate in experimental studies, i.e., the lack of capacity to consent, the need for taking confounding medication, the required but disturbing support by relatives and caregivers. The situation becomes even worse when it comes to studies in brain imaging scanners for e.g., during Magnetic Resonance Imaging (MRI) due to claustrophobic arousal, further limitations of communication between participant and investigator and “incomprehensible” technical procedures and environment. Iversen et al. give in their review article “*Promoting successful participation of people living with Alzheimer’s disease and related dementias in pain-related neuroimaging research studies”* a lot of information about these problems and their solutions to ease brain imaging studies in persons with dementia.

Pain has appeared to have at least two dimensions, namely a sensory and an affective dimension, with distinct neural circuits in the spine and in the brain. There is common belief that pain-related distress and suffering are mainly consequences of the activation of the affective dimension. Hence, the affective dimension especially asks for pain-reducing treatment. Anderson et al. report in the present article “*Increased pain unpleasantness and pain-related fMRI activation in the periaqueductal gray in Alzheimer’s disease”* that persons with Alzheimer dementia had a tendency to more pain-related unpleasantness when experimentally stimulated. Furthermore, they present data suggesting that an over-activation of the periaqueductal grey (PAG) occurs during pain, a brain center full with opioid receptors controlling endogenous analgesia, and might be reason for more pain unpleasantness. Thus, a midbrain problems may let persons with dementia suffer more than healthy people when in pain.

Our third thematic topic relates to individual differences in persons with dementia, which may modulate the effects of dementia on pain. There are meanwhile several studies considering age and gender as modulating factors; however, race and ethnicity have yet been understudied. Therefore, it is timely to present a study on the potential differences between White and Black people living in senior residences in expressing clinical pain when assessed by the Pain Assessment in Advanced Dementia (PAINAD). Resnick et al. report in their present article on the “*Invariance of the PAINAD scale between the black and white residents living with dementia”*. For this conclusion, they have used several parameters of reliability and validity.

Altogether, we could fill this Research Topic on pain and dementia by five excellent up-to-date contributions of how overt and cerebral behaviors contribute to changed pain processing in persons with dementia, also considering race and ethnicity as individual factor.

